# 

**Published:** 1900-04

**Authors:** 


### INDEX TO VOLUME II.

Abdomen, Penetrating Wounds of, etc ........ 88
Adamless Eden, An ........ 637
African Remedy for Dysentery ........ 504
Address to Grady Hospital Nurses ........ 306
Alimentary Canal of Infants, Fermentative Disorders of ........ 92
American Medical Association ........ 253
American Text-Book of Physiology ........ 530
American Year Book of Medicine and Surgery, The ........ 62, 126, 842
Ammoniated Tr. of Gum Guaiac ........ 663
Amputation and Longevity ........ 210
Anatomy and Histology of the Mouth and Teeth ........ 635
Anatomy of the Brain, The ........ 462
Anatomical Science among Ancient Egyptians ........ 682
Ankle Sprains ........ 642
Ankylosis ........ 41
Ankylostomiasis the Cause of Puerto Rican Fever ........ 128
Annual of Eclctic Medicine and Surgery ........ 635
Army Canteen, Abolition of ........ 424
Atlanta College of Physicians and Surgeons ........ 115
Atlanta Medical Society, Officers of ........ 686
Atlas and Epitome of Diseases Caused by Accidents ........ 401
Atlas of Legal Medicine ........ 125
Attention Called to Valuable Remedy ........ 742

Bacteriology and Surgical Technique for Nurses ........ 695
Birth-rates, Alcoholism, etc ........ 853
Boil and Abscess Cavities, Rapid Method of Healing ........ 34
Book of Diet Lists, A ........ 767
Bourns, Dr. Frank S. ........ 679
Brain Traumatisms ........ 145
Brer Rabbit and the Tar Baby ........ 186
Bromides in Hysteria, Use of ........ 863
Bubonic Plague, The ........ 208

Cancer Cases, How Far is the Surgeon Justified in Operating on Hopeless? ........ 349
Catarrhal Conjunctivitis, Treatment of ........ 359
Catarrhal Deafness ........ 427
Catarrhal Deafness, Electrolysis in Treatment ........ 815
Causes of Pains in the Feet ........ 329
Cerebro-Spinal Fever ........ 713
Ceremonies among Hottentots ........ 829
Chairman's Address ........ 95

Chicago Drainage Canal........ 432
Cholera Infantum........ 249
Cholera in the East........ 647
Chloretone in Cholera Morbus........ 447
Christian Science—An Exposition........ 127
Christian Science Bubble, The........ 316
Chronic Dyspepsia Treated with $H_2O_2$........ 67
Climate of Puerto Rico, The........ 73
Clinical Examination of Urine........ 262
Clinical Examination of Urine and Urinary Diagnosis........ 695
Clinical Society of Maryland........ 666
Cocaine Sniffing........622, 746
Cold in Typhoid Fever, Way to Employ........ 213
Conservative Tendency in Genito-Urinary Work........ 464
Continued Use of Antiseptic and Eliminative Treatment in Typhoid Fever........ 298
Cyclic Albuminaria in Adolescents........ 720
Cyclopedia of Practical Medicine, A........ 128

Danger to Eye while Chopping with a Hoe........ 311
Dangers of Vaginal Irrigation........ 422
Deafness in Children........ 72
Defeat of the Osteopathy Bill, The........ 685
Determination of Sex........ 431
Diabetes Mellitus........ 505
Diabetes Mellitus and Glycosuria, On........ 531
Diabetes Mellitus, Successful Treatment of........ 65
Diagnosis and Etiology of Idiopathic Diseases........ 367
Diagnosis, Essentials of........ 196
Digitalis in Treatment of Heart Diseases........ 501
Discovery of Ureine........ 709
Diseases of Skin, Essentials of........ 196
Diseases of the Stomach........ 226
Disinfection, Modern Methods in........ 481
Doctor's Income, The........ 347
Does "Cross-Eye" Affect the General Health........ 340
Duane's Medical Dispensary........ 765

Economy in Employment of Cocaine........ 791
Editor and the Advertiser........ 621
Egbert's Hygiene........ 692
Electricity in Medicine........ 437
Epigæa for Eructation........ 496
Ergot in Diseases of Prostate, Value of........ 246
Essential Hemorrhage from Kidneys........ 272
Essentials of Hematology........ 328
Excision of External Carotid Artery........ 609
Eye, Diseases of........ 328
Eye, Ear, Nose and Throat........ 634

Female Breast, Diseases of .......... 565
Fireman's Cramp .......... 408
Formalin as an Antiseptic .......... 500
Follicular Tonsillitis .......... 858
Fractures, Treatment of .......... 463
French Surgeon of the Old School .......... 275

Gall-stones and Diseases of Gall-bladder .......... 400
General Therapeutical Action of Electricity .......... 535
Georgia Medical Association, The .......... 117
Georgia Pasteur Institute .......... 624
Gonorrhea, Recent Observations on Treatment of .......... 737
Gonorrhea, Treatment of .......... 784

Heart, Diseases of .......... 767
Heart in Life Insurance, The .......... 1
Hemorrhage occurring after Menopause .......... 172
Hemorrhage, Simple Operation for .......... 647
Histology, Text-Book of .......... 636
Histology, Text-Book of .......... 693
How to Live a Century .......... 278
Husband's Liability for Care of Insane Wife .......... 645
Hydrophobia Institute Elects Officers .......... 502
Hydrozone and Glycozone in Gastric, etc .......... 574
Hygiene and Sanitation .......... 636
Hypnotic Action of Apomorphine .......... 267

Imperialism and Porto Rico Smallpox .......... 432
Index to the Character of Medical Journals, An .......... 684
Indications for and Method of Operating upon the Middle Turbinated Bone .......... 6
Indications for Operation upon Liver and Biliary Passages .......... 417
Inebriety, Medical Treatment of .......... 99
Infantile Convulsions, Treatment of .......... 444
Infantile Scurvy .......... 358
Inflammation, Treatment of .......... 107
Injuries to Eye in Medico-Legal Aspect .......... 195
In Lightning Vein .......... 420
Inside Information .......... 211
Is the Little Toe Disappearing? .......... 521
Intermittent Pulse in Life Insurance Examination .......... 137
International Text-Book of Surgery .......... 59, 127

King's American Dispensary .......... 766
King's Manual of Obstetrics .......... 401
Kleen on Diabetes .......... 531

Laboratory and Percentage Feeding of Infants .......... 335
Lactation in a Virgin .......... 214
Laparotomy for Abdominal Wounds during War .......... 426
Last Words of Meiamoun .......... 212

Latent Gonorrhea in Male .......... 21
Later Sequelæ of Accidents during Parturition .......... 238
Law and the Doctor, The .......... 827
Lectures on Principles of Surgery .......... 328
Legal Prevention of Tuberculosis .......... 406
Leprosy, Danger from .......... 140
Lessons on Anatomy, Physiology and Hygiene of Infants .......... 841

Management of Acute Diarrheas of Infants .......... 164
Management of Labor .......... 793
Manual of Materia Medica and Pharmacology .......... 636
Manual of Modern Surgery .......... 62
Manual of Personal Hygiene .......... 462
Manual of Practice of Medicine, A .......... 62
Masturbation, Physiological Therapeutics of .......... 372
Materia Medica and Therapeutics .......... 63
McGuire, Hunter, Death of .......... 448
Medical Annual and Practitioners' Index, The .......... 261
Medical Defense Union .......... 256
Medical Diseases of Infancy and Childhood .......... 693
Medical Gymnastics .......... 649
Medical Missionary .......... 348
Medical News Visiting List .......... 694
Medicine in Fiction .......... 754
Medico-Legal Questions .......... 345
Membranous Croup .......... 659
Menorrhagia in Young Girls .......... 201
Mercurol in the Treatment of Gonorrhea .......... 344
Metric System Again, The .......... 199
Microtomist's Vade-Mecum .......... 636
Mississippi Valley Medical Association .......... 386
Modern Business Methods for Medical Profession .......... 263
Modern Methods of Disinfection .......... 481
Modern Treatment of Diabetes .......... 503
Modern Treatment of Incipient Pulmonary Tuberculosis .......... 543
Mosquito Family, The .......... 792
Music .......... 569

Navel, Treatment of the .......... 357
Neuralgia, Treatment of .......... 313
News Items .......... 430
News Items Pan-American Congress .......... 675
Non-malignant Gastric Ulcers, Treatment of .......... 144
Normal Histology .......... 261
Nose and Throat, Diseases of .......... 125
Notable Gains in Materia Medica, Some .......... 788
Notes on Plague in China and India .......... 285
Notice to Subscribers .......... 674

Office Dispensing........ 494
Operative Surgery (Stimson)........ 400
Ophthalmic Therapeutics........ 243
Osteo-Arthritis and Rheumatoid Arthritis........ 577
Osteopathy again........ 523
Overeating........ 288

Pain........ 361
Pan-American Exposition........ 386
Panama and the Sierras........ 764
Panama Fever........ 69
Park's, Dr., Article........ 454
Passing Notice of Psychology........ 433
Pathological Specimens........ 28
Photo-Therapy........ 640
Physical Culture in Medical World........ 361
Physician's Visiting List........ 694
Physiological Therapeutics of Borderland Insanity........ 597
Physiological Therapeutics Dypsomania........ 724
Plague, The........ 320
Plugging Uterus in Abortion........ 270
Pneumonia in Children........ 847
Pocket Medical Dictionary, A........ 62
Points in Surgery........ 429
Polk's Medical Register........ 462
Practical Anatomy (Eckley)........ 126
Practical Diagnosis in Obstetrics........ 842
Practical Medicine (Thomson)........ 461
Practical Treatise on Sexual Disorders........ 196
Practical Uranalysis (Purdy)........ 461
Premature Burial........ 388
President's Address........ 721
Principles of Treatment (Bruce)........ 64
Progressive Medicine........ 695
Progressive Pernicious Anemia........ 207
Propriety........ 353
Prostatic Hypertrophy, Treatment of........ 856
Prostato-cystitis........ 383
Pruritus Ani........ 411
Psychoses of Puberty, The........ 573
Pulmonary Tuberculosis (Knopf)........ 530

Recent Opinions on General Paralysis of Insane........ 350
Refraction of Eye, The........ 26
Relation of Profession to General Public........ 793
Religious Psychology........ 515
Richmond Academy of Medicine........ 36
Rickets, Treatment of........ 352
Reminiscences of a Physiologist........ 696

Review of Recent Legal Opinions on Physicians .......... 636
Rhinology, Laringology and Otology .......... 634
Rudiments of Modern Electricity .......... 766

Scarlet Fever, Treatment of .......... 338
Section on Ophthalmology .......... 616
Sequelæ of Scarlet Fever .......... 843
Sexual Debility in Man (Sturgis) .......... 692
Shall the Specialist Divide Fee? .......... 342
Shall We Tell Women with Uterine Cancer the Nature of their Disease? 486
Shock and Its Surgical Significance .......... 356
Sickness in High Altitude .......... 825
Sleeplessness .......... 205
Smoke Nuisance, The .......... 453
Some of Accidents from Obstetric Forceps .......... 773
Some Experiences in the South African War .......... 543
Sour Lake, Texas .......... 13
Southern Surgical and Gynecological Association .......... 611
Spinal Cord Cocainization .......... 520
Splenectomy for Congestive Hypertrophy .......... 289
Standardization of Drugs .......... 643
Stricture of Rectum, Treatment of .......... 268
Studies in Sex Psychology .......... 531
Suggestions on Puerperal Eclampsia .......... 233
Suggestions on Use of Protargol .......... 864
Sulphate of Magnesia .......... 510
Surgical Fanaticism .......... 428
Surgical Pathology .......... 61
Suspending Trial for Examination by Physicians .......... 646
Symptomatologic Diagnosis of Valvular Obstipation .......... 414
Syphilis among Alaskan Indians .......... 491
Syphilis, Treatment of .......... 861
System of Practical Therapeutics (Hare) .......... 841

Tarnier's Monument .......... 504
Text-Book on Diseases of Women .......... 262
Text-Book of Histology .......... 693
Text-Book of Pathologic Bacteria .......... 693
Text-Book of Practical Obstetrics .......... 841
Text-Book of Practice of Medicine (Anders) .......... 693
Text-Book of Practice of Medicine .......... 693
Tirard's Medical Treatment .......... 327
Transactions American Orthopedic Association .......... 766
Treatise on Diseases of Nose and Throat .......... 633
Treatment of Influenza .......... 850
Tri-State Medical Society Georgia, Alabama, Tennessee .......... 390
Tuberculosis, Early Diagnosis of .......... 355
Typhoid Fever .......... 176
Typhoid Fever in Young .......... 203

Unna's Dressing .... 498
Urinary Diagnosis and Test .... 765
Urine of Horse in Pneumonia .... 744
Use of Bromides in Hysteria .... 863
Uterine Douche, The .... 134

Vacancies to be filled in U. S. M. H. S .... 143
Vomiting of Pregnancy, Treatment of .... 402
Vomiting, Surgical Aspect of .... 276

Western Ophthalmologic Association .... 111
What Vermouth is made of .... 197
Why I Use Pepto-Mangan .... 214
Women, Diseases of .... 764

X-Ray Diagnosis of Calculus .... 201

Year Book of Nose and Throat and Ear .... 60

(VIII)

### CONTRIBUTORS TO VOLUME II.

Arnold-Snow, Mary L. H. ... 361
Bate, B. Alex ... 505
Bovee, J. W. ... 289
Brantley, F. M. ... 367
Bullard, W. L. ... 311
Calhoun, A. W. ... 243
Chatfield, A. E. ... 249
Church, W. F. ... 107
Corson, E. R. ... 246
Crother, T. D. ... 99
Davidson, J. P. ... 6
Davis, E. C. ... 172
Day, J. B. H. ... 383
Frazier, L. G. ... 92
Hall, Rufus B. ... 28
Hardman, W. B. ... 176
Hardon, V. O. ... 306
Henson, J. W. ... 381
Hoge, M. D., Jr. ... 744
Hoke, Michael ... 577
Howard, N. F. ... 510, 663, 742
Hubbard, T. V. ... 298, 721
Hutchins, M. B. ... 34
Kenner, R. C. ... 313
Kime, R. R. ... 233, 793
Leitch, T. C. ... 737
Lindorme, C. A. F. ... 316, 372, 597, 724
Nicolson, W. P. ... 609
Oertel, T. E. ... 217
Parks, W. B. ... 433
Proben, Chas. J. ... 73
Reed, E. B. ... 447
Robinson, Gilman P. ... 164
Roy, Dunbar ... 815
Smith, W. Monroe ... 799
Smithwick, J. W. P. ... 444
Snow, W. B. ... 437
Spratling, E. J. ... 226
Stevens, E. S. ... 238
Stucky, J. A. ... 95
Taylor, Hugh M. ... 88
Toepel, Theodore ... 649
Upshur, J. D. ... 1
Visanska, S. A. ... 659
Wheat, Lewis ... 21
Wilkinson, C. H. ... 13
Williams, Howard J. ... 145
Wolff, Bernard ... 515

**This Journal and Atlanta Semi-weekly Journal one year for $1.00 cash in advance. No checks.**

**This Journal and Atlanta Semi-weekly Journal one year for $1.00 cash in advance. No checks.**

**This Journal and Atlanta Semi-weekly Journal one year for $1.00 cash in advance. No checks.**

***NOTICE.–This JOURNAL and THE INTERNATIONAL JOURNAL OF SURGERY one year for $1.00 cash, to new subscribers. No out-of-town checks accepted unless 10c. is added for cost of cashing.***

---

SHEER SELFISHNESS.

"Her husband died of heart failure."

"Oh, dear, how selfish! Why couldn't he have been ill a couple of months or so, that she might have had a chance at that lovely young doctor nearby. But some men are so thoughtless."—*Ex.*

---

**This Journal and Atlanta Semi-weekly Journal one year for $1.00 cash in advance. No checks.**

***NOTICE.—This JOURNAL and THE INTERNATIONAL JOURNAL OF SURGERY one year for $1.00 cash, to new subscribers. No out-of-town checks accepted unless 10c. is added for cost of cashing.***

**This Journal and Atlanta Semi-weekly Journal one year for $1.00 cash in advance. No checks.**

**This Journal and Atlanta Semi-weekly Journal one year for $1.00 cash in advance. No checks.**

**This Journal and Atlanta Semi-weekly Journal one year for $1.00 cash in advance. No checks.**

---

### AN INSTRUMENT OF PRECISION.

"What is a stethoscope and what is it used for?" asked the professor of the class in anatomy.

"The stethoscope," answered the pupil at the pedal extremity of the class, "is a sort of microscope used by a doctor for the purpose of looking into the chest of a patient with his ear."—*Chicago News,*

---

***NOTICE.—This JOURNAL and THE INTERNATIONAL JOURNAL OF SURGERY one year for $1.00 cash, to new subscribers. No out-of-town checks accepted unless 10c. is added for cost of cashing.***

